# Intramedullary nailing vs modular megaprosthesis in extracapsular metastases of proximal femur: clinical outcomes and complication in a retrospective study

**DOI:** 10.1186/s12891-022-05728-5

**Published:** 2022-09-13

**Authors:** Raffaele Vitiello, Carlo Perisano, Tommaso Greco, Luigi Cianni, Chiara Polichetti, Rocco Maria Comodo, Ivan De Martino, Vincenzo La Vergata, Giulio Maccauro

**Affiliations:** 1 Fondazione Policlinico Universitario Agostino Gemelli - IRCCS, Largo Agostino Gemelli 8, 00168 Rome, Italy; 2 Università Cattolica Del Sacro Cuore, Rome, Italy

**Keywords:** Metastasis, Proximal femur, Trochanteric, Megaprosthesis, Nailing, NLR, PLR

## Abstract

**Background:**

Extracapsular proximal femur metastasis could be treated by synthesis or resection and megaprosthesis. No universal accepted guidelines are present in the literature. The aim of our study is to analyze of patients with metastases in the trochanteric region of the femur treated by a single type of intramedullary nailing or hip megaprosthesis.

**Methods:**

We retrospectively reviewed all patients affected by extracapsular metastases of proximal femur. Anthropometric and anamnestic data, routine blood exams and complications were collected. VAS score and MSTS score was administered before the surgery, ad 1–6-12 months after surgery. An un-paired T test and Chi-square were used. Multiple linear regression and logistic regression was performed. Significance was set for *p* < 0.05.

**Result:**

Twenty patients were assigned in intramedullary Group, twenty-five in megaprostheses Group. The mean operative time is shorter in intramedullary group. Differential shows a higher anemization in megaprostheses group (2 ± 2 vs 3.6 ± 1.3; *p* = 0.02). The patients of intramedullary group showed malnutrition (Albumin: 30.5 ± 6.5 vs 37.6 ± 6 g/L; *p* = 0.03) and pro-inflammatory state (NLR: 7.1 ± 6.7 vs 3.8 ± 2.4; *p* = 0.05) (PLR: 312 ± 203 vs 194 ± 99; *p* = 0.04) greater than megaprostheses group. The patients in intramedullary groups shows a higher functional performance score than megaprostheses group at 1 month follow-up (MSTS: 16.4 ± 6.3 vs 12.2 ± 3.7; *p* = 0.004). A multivariate analysis confirms the role of type of surgery (*p* = 0.001), surgery duration (*p* = 0.005) and NLR (*p* = 0.02) in affecting the MSTS.

Globally eight complications were recorded, no statistical difference was noticed between the two groups (*p* = 0.7), no predictor was found at logistic analysis.

**Conclusion:**

Intramedullary nailing guarantees a rapid functional recovery, compared to patients undergoing hip megaprosthesis who instead improve gradually over time. The selection of patients with poor prognosis allows the correct surgical indication of nailing, while in the case of a more favorable prognosis, the intervention of hip megaprosthesis is to be preferred.

## Background

The femur is one of the most frequent sites of osseous metastatic lesions [1]. Approximately 10% of patients with primary malignant tumors will develop metastases of the proximal femur [2]. Concerning the proximal femur, about half of the lesions occur in the femoral neck, 30% in the subtrochanteric region and 20% in the intertrochanteric site.

Femoral metastases is a frequent cause of morbidity and mortality in patients with advance-stage cancer and often surgery is indicated and required [3]. The decision to operate and the technique choice need a multidisciplinary team meeting that analyzes multiple patients related factors [4–7] as general health status, locoregional extension of the malignancy and life expectancy [4, 5].

Femoral intertrochanteric and subtrochanteric region require some special considerations: the biomechanically forces and the critical vascularization [8] associated to the poor healing percentage, make it difficult to choose the proper pathological fractures surgical treatment [9].

The two surgical options for the management of proximal femur pathological fractures are: resection and reconstruction by modular prosthesis or standard arthroplasty; reduction and fixation through the use of an intramedullary nail, or a screw-plate or cement supported by further synthesis [2, 10, 11].

Intramedullary nailing (IN) stands out for simplicity, low cost, simpler technique and limited dissection, but yet it does not guarantee the local control of the disease. On the other hand, resection and reconstruction by megaprosthesis allows a better and lasting function but longer surgical time, higher costs and complications [12] such as dislocation and infection [12–14].

Numerous previous papers have reported strategies and algorithms to decide which treatment best adapts to every patient, however the topic is still under debate [15–18]. Patients with proximal femur metastases present with a wide variability of factors that make analysis and indexing overly difficult [19]. The Literature on this subject is highly controversial and comes with a very inhomogeneous population (including femoral head, neck and trochanteric metastases), different synthesis or prostheses and rarely use clinical evaluation scores or hematological and blood chemistry parameters.

Furthermore, some papers have tried to analyze the trends on the surgical treatment of pathological proximal femur fractures underlining the lack of accepted guidelines stressing the need of evidence-based consensus and prospective randomized studies to develop consistent and evidence-based treatment recommendations [20].

A moderate-sized lesion with a normal bone in the femoral head and no neck fracture commonly are indication for reduction and synthesis [21]. In all other cases, modular endoprosthesis or arthroplasty are the option to choose.

The aim of our study is to analyze the clinical and laboratory outcomes and complications of patients with metastases in the trochanteric region of the femur treated by a specific type of intramedullary nail (IN) or hip megaprosthesis (HM).

## Methods

We retrospectively reviewed all patients admitted from January 2016 to December 2020 to our Orthopaedic Department affected by trochanteric metastases of the proximal femur.

Inclusion criteria were: trochanteric metastases of the proximal femur, Intramedullary nailing or hip megaprosthesis implantation, informed consent to participate in the studies.

Exclusion criteria were: age under 18 years old, follow-up less than 6-months.

The trochanteric region was defined from the base of the neck, greater and lesser trochanter and up to 5 cm below the lesser trochanter.

Patients were divided in a IN Group, for those who underwent Intramedullary nailing in case of poor prognosis and in a HM Group, for hip megaprostheses implantation in case of better prognosis, evaluated for each case by a multidisciplinary board.

### Surgical technique

All the procedures were performed by three orthopaedic surgeons fellowship-trained in oncological surgery.

A general anesthesia was performed in all cases. All patients received Cephazoline 2 g i.v. as antibiotic prophylaxis before surgery, if not contraindicated [22]. A urinary catheter was placed in all patients and removed within 72 h after the surgery.

Patients in IN Group were placed in supine decubitus on traction bed. A lateral approach was used. Close reduction and internal fixation were performed, long intramedullary nail was implanted to arm all the femur according to the manufacturer technique (PFNA long; Depuy Synthes) [23]. Intramedullary spiral blade and distal antirotational screws were implanted. Rimming wasn’t ever needed. No drainage was placed.

Patients in HM Group were placed in lateral decubitus position. A lateral approach was used. After bone exposure, an en block resection was performed, cementless silver-coated megaprosthesis was implanted according to the manufacturer technique (Mutars; Implantcast) [24, 25]. Silver-coated megaprosthesis was preferred in order to reduce risk of post-surgical infections. No acetabular components were implanted. The myodesis through the Trevira Tube © (Implantcast; GmbH, Buxtehude, Germany) completed the surgery [26, 27]. One intra-articular closed-suction drainage was placed and then removed 48 h after surgery.

All patients followed the same post-operative rehabilitation protocol: at 48 h after surgery patients were seated with their feet out of bed; at 72 h, they were allowed to progressive weight bearing with walker frames. Walking without aids was achieved in two months.

Patients were regularly followed-up at 2 and 4 weeks after surgery and then every 3 months for the first two years, then yearly. Starting 4 weeks after surgery, an x-ray was performed at each clinical evaluation.

### Clinical evaluation

Anthropometric and anamnestic data and routine blood exams were collected.

Routine blood exams were used to calculate Neutrophil–Lymphocyte Ratio (NLR) and Platelet-Lymphocyte ratio (PLR) [28, 29]. NLR is normal below 3 points showing mild to severe inflammation for growing scores.

Karnofsky performance score [30], Musculoskeletal Tumor Society (MSTS) scoring system [31] and pain evaluation through Visual Analogue Scale (VAS) score [32] were administered to all patients at hospital admission picturing the pre-surgery status and then at 1 month, 6 months and 12 months follow-up.

VAS score ranged from 0 point, for no-pain, to 10 points for the worsted pain ever felt.

Karnofsky performance score ranged from 0%, a death patient, to 100%, completely self-sufficient and asymptomatic patient [30].

MSTS score take into account six different area: pain, function, emotional, supports, walking and gate. Each area is awarded by a maximum of five points in the case of the best result. MSTS score ranged from 0 to 30 points [31].

During the hospitalization and outpatient follow-up all the complications were recorded (wound dehiscence, deep infection, painful local progression, dislocation, deep thrombosis, pulmonary emboli, severe anemia, pneumonia and urinary tract infection). Wound dehiscence or surgical site infection was defined as a delayed of normal healing of the surgical wound with presence of redness, edema and secretion in absence of deep tissue involvement or general symptoms [33].

### Radiological assessment

Fractures or impending fracture were diagnosed through a standard X- ray series and a computer tomography (CT) in all cases. Mirel score was used to stratify the fracture risk [34]. For a Mirel score over 7 points surgical indication was confirmed [34].

### Data analysis

GraphPad QuickCalcs (GraphPad Software, San Diego) was used for data analysis. The data were reported as mean and standard deviation (± SD).

The asymmetry was calculated to evaluate the normality of the different parameters.

A paired T test was performed for pre and post-operative comparison of MSTS. An un-paired T test was used to compare anthropometric, anamnestic data, Karnofsky, MSTS, VAS, blood exams between groups. Chi-square test was performed for evaluation of complication in the two group. Multiple linear regression was used to match the functional outcomes and complication’s incidence in the population study. Logistic regression was performed to analyze the odds ratio of different parameters in the incidence of complication. Significance was set for *p* < 0.05.

## Result

One hundred twenty-nine patients were affected by trochanteric metastases during our analysis period.

Forty-five patients were considered eligible according to the inclusion and exclusion criteria and were finally enrolled in the study.

Twenty patients were assigned in the IN Group and twenty-five in the HM Group.

The primary outcome analyzed was the post-surgery clinical outcomes (MSTS and VAS score). The secondary outcome was blood exams evaluation and incidence of complications.

There were 16 male and 29 female, the mean age was 68.2 years old (± 10); the mean BMI was 25.4 points (± 3.7). The mean follow-up was 21.2 months (± 16.3). No statistical differences were noticed between IN Group and HM Group concerning the age, gender, BMI or active fracture/impending fracture (Table 1).

**Table 1:** Baseline characteristics

	**TOTAL**	**INTRAMEDULLARY NAILING GROUP**	**HIP MEGAPROSTHESIS GROUP**	**P**
PATIENTS	45	20	25	
SEX	16 M | 29 F	6 M | 14 F	10 M | 15 F	
AGE (YEARS)	68.2 ± 10	69.4 ± 10	67.5 ± 9.9	0.5
BOBY MASS INDEX	25.4 ± 3.7	24.9 ± 4.1	25.4 ± 3.5	0.1
SKELETAL METASTASES	22	12	20	
VISCERAL METASTASES	13	10	3	
ACTIVE FRACTURE	21	8	13	
IMPENDING FRACTURE	24	12	12	
HEMOGLOBIN PRE-SURGERY (G/DL)	12 ± 1.8	11.3 ± 1.8	12.6 ± 1.7	0.2
HEMOGLOBIN POST-SURGERY (G/DL)	9.3 ± 1.3	9.9 ± 1.7	8.9 ± 0.8	0.2
DIFFERENTIAL HEMOGLOBIN (G/DL)	2.7 ± 1.9	1.4 ± 1.8	3.6 ± 1.3	0.02*
ALBUMINE (G/L)	33.2 ± 7.1	30.6 ± 6.5	37.2 ± 6.3	0.03*
NLR	5.2 ± 4.9	7.1 ± 6.7	3.8 ± 2.4	0.05*
PLR	245 ± 161	312 ± 203	195 ± 99	0.04*
FOLLOW-UP (MONTHS)	21.2 ± 16.3	9 ± 5.4	30.5 ± 15.9	0.001*

In brackets measurement unit; Data were reported as absolute value ± SD. * underline statistical significance

Breast cancer was the most common primary tumor (33%), followed by lung (22%), myeloma (20%), prostate (11%), kidney (8%) and thyroid (6%). In the IN Group were more frequent lung cancer (7 vs 3 patients), while in contrast in the HM Group breast cancer (10 vs 5 patients) and prostate cancer (5 vs 0 patients) were prevalent.

In the IN Group preventive surgery was more frequent than HM Group, but without statistical difference (60% vs 48%; *p* = 0.2). Mean resection in the HM Group was 11.7 ± 3 cm.

In the IN Group additional skeletal metastases were found in 12 patients (60%), mostly located in the pelvis and spine; 10 patients (50%) also showed visceral metastases. In the HM Group, however, further bone lesions were present in 20 patients (80%), but only 3 had visceral metastases (12%).

The mean Karnofsky score was 76% (± 21), with no difference through the IN Group and the HM Group (*p* = 0.9). The average Karnofsky score indicates that patients are unable to do effort or work but are able to take care of themselves.

The mean operative time is shorter in the IN Group than the HM Group (105 ± 49 vs 159 ± 37 min; *p* = 0.0001), as expected.

No difference was noticed among hemoglobin before and after surgery, white blood cell and platelet (Table 1). Differential hemoglobin (post-surgery minus prior surgery) shows a higher acute anemia in the HM Group (2 ± 2 vs 3.6 ± 1.3; *p* = 0.02).

The patients of the IN Group showed instead malnutrition (Albumin: 30.5 ± 6.5 vs 37.6 ± 6 g/L; *p* = 0.03) and pro-inflammatory state (NLR: 7.1 ± 6.7 vs 3.8 ± 2.4; *p* = 0.05) (PLR: 312 ± 203 vs 194 ± 99; *p* = 0.04) greater than the HM Group.

**Fig. 1:**
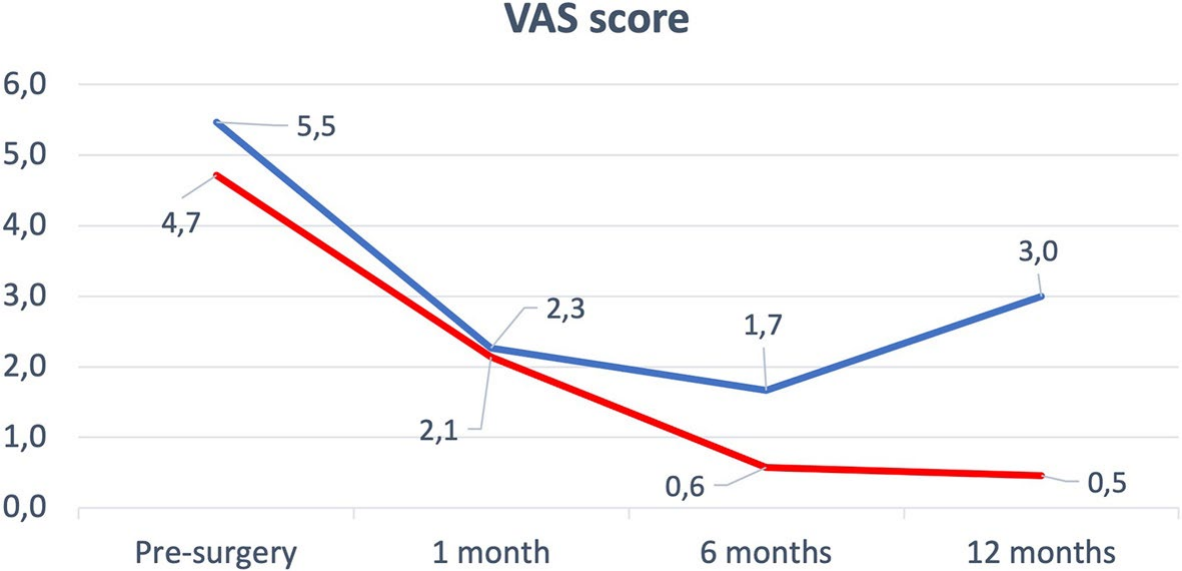
VAS Score. VAS score trend at the various follow-ups. Blue line for IN group, red line for HM group

VAS score pre-surgery and at 1–6 months after surgery didn’t show differencees between the two groups (Table 2)(Fig. 1).

**Table 2:** Clinical outcomes

	**TOTAL**	**INTRAMEDULLARY NAILING GROUP**	**HIP MEGAPROSTHESIS GROUP**	**P**
PATIENTS	45	20	25	
KARNOFSKY PRE-SURGERY	7.5 ± 2.2	7.6 ± 2.6	7.6 ± 1.7	0.9
VAS PRE-SURGERY	5.2 ± 4.1	5.4 ± 4.3	4.7 ± 4.2	0.6
MSTS PRE-SURGERY	15 ± 9	15.7 ± 9	15.1 ± 10	0.8
VAS 1 MONTH	2.1 ± 2	2.3 ± 1.8	2.1 ± 2.2	0.8
MSTS 1 MONTH	14.1 ± 5.4	16.4 ± 6.4	12.3 ± 3.7	0.04*
VAS 6 MONTHS	0.9 ± 1.4	1.7 ± 1.8	0.5 ± 1.2	0.1
MSTS 6 MONTHS	18.5 ± 4	16.8 ± 6.2	19.2 ± 2.4	0.2
VAS 12 MONTHS	1 ± 1.8	3 ± 3	0.5 ± 0.8	0.01*
MSTS 12 MONTHS	18.6 ± 5.5	17 ± 5.7	19.1 ± 5.6	0.5

In brackets measurement unit; Data were reported as absolute value ± SD. * underline statistical significance

**Fig. 2:**
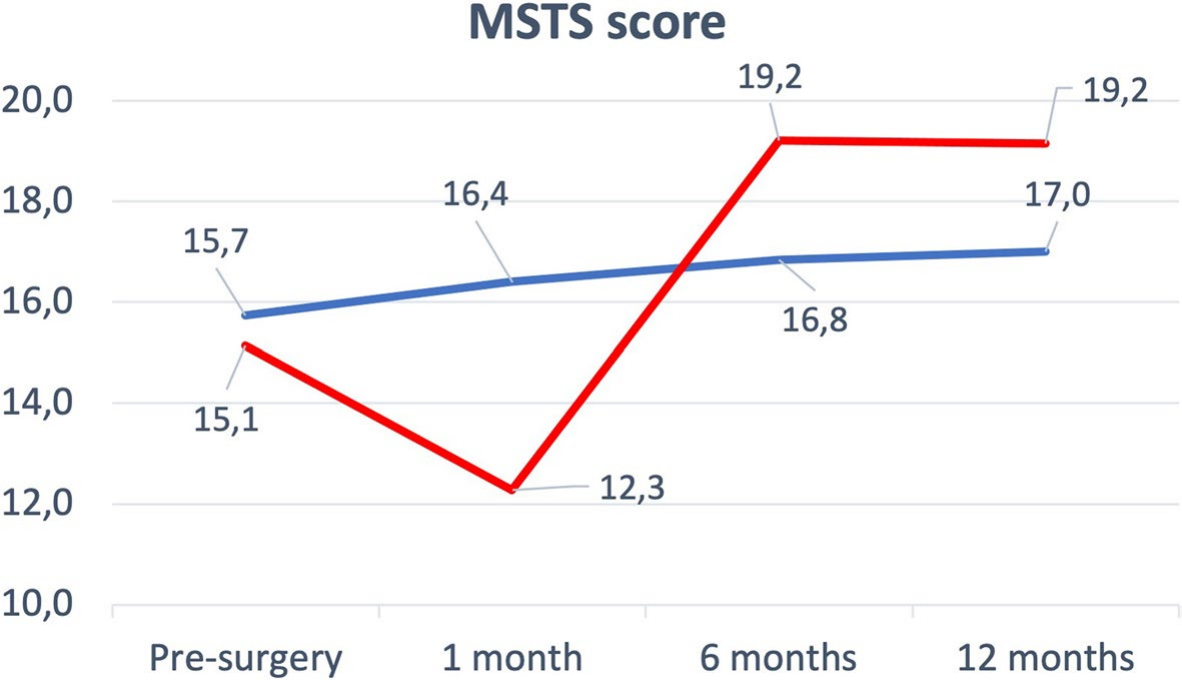
MSTS Score. MSTS score trend at the various follow-ups. Blue line for IN group, red line for HM group

At 12-months follow-up the IN Group showed more pain than the HM Group (3 ± 3 vs 0.5 ± 0.8; *p* = 0.01). MSTS pre-surgery, at 6 and 12 months follow-up didn’t show differences (Table 2)(Fig. 2). Only at 1-month follow-up the MSTS score was higher in IN Group than HM Group (16.4 ± 6.3 vs 12.2 ± 3.7; *p* = 0.004).

Comparing the MSTS results within the two groups at the various follow-ups the improvement in the HM Group between 1 and 6 months after surgery was statistically significant (12.3 ± 3.7 vs 19.2 ± 2.4; *p* = 0.001).

Globally eight complications were recorded. In the IN Group (3/20; 15%) one superficial wound infection (1/20 patients; 5%), one pneumonia (1/20 patients; 5%) and one nail breakage (1/20 patients; 5%) were recorded. In the HM Group instead (5/25; 20%) two cystitis (2/25 patients; 8%), two pneumonia (2/25 patients; 8%) and one dislocation (1/25 patients; 4%) were recorded. No statistical difference was noticed between the two groups (*p* = 0.7).

All the patients affected by infections were successfully treated by antimicrobial oral therapy with complete resolution with no need of further surgery. The dislocation was treated by open reduction and implantation of acetabular cup and bis mobility system. Nail breakage was a challenging complication [35] treated by resection and reconstruction with HM.

A multivariate analysis was performed matching the MSTS score with type of surgery, surgery duration, age of patients and NLR. The analysis confirms the statistical significance of type of surgery (*p* = 0.001), surgery duration (*p* = 0.005) and NLR (*p* = 0.02).

Regarding complication a logistic regression was performed to analyze the odds ratio of age, surgery duration, type of surgery, BMI and NLR, but none of parameters reach the significancy.

## Discussion

The surgical treatment of proximal femur metastases is a hot topic for oncological surgeons still quite discussed. The recent literature shows different article in favor of hip replacement [2, 36], other show similar result between the IN and the HM [15, 37, 38], while still others prove the safety of the IN [39].

Unfortunately the literature on this topic present many bias: first of all the use of different devices in the same study [2, 40, 41] (such as IN, HM, standard hip prosthesis, plate or screw-plate) and to complicate the scenario also the presence of different settings among the same device [15]. In many studies there are tons of different surgeons [40], different areas of the femur involved [41, 42], different primary tumors and rarely clinical outcomes collection [2, 43–46]. Furthermore, as underlined in the Chafey work [21], the high mortality in these patients represent a confounding factor that could distort the incidence and then the analysis of other factors.

Fakler et al. reported in a similar population a very lower survival rate of the patients in comparison with our findings probably due to the advanced disease highlighted by the poor Karnosky index [15]: 6.5 points in the HM group (vs 7.5 in our work) and 4.5 in the IN group (vs 7.6 in our work). As already seen, our data do not show a substantial difference in the Karnofsky score between the patients in the groups under examination, while instead there is a substantial difference regarding NLR and PLR values. These latter have proved to be important negative prognostic factors in oncological patients [28, 29], underlining how the IN group starts from a poor prognosis condition.

Another factor implicated in better survival and outcomes seemed to be preventive intervention in impending fractures [19, 47]. Compared to the results presented by Mavrogenis et al. [19], this trend is not confirmed in our population and preventive intervention does not appear to impact the functional outcomes reducing the risk of complications.

There are a few studies that take the clinical outcomes into consideration [2, 43, 44]. In particular. Guzik et al. showed low MSTS pre-operative values (HM 6.4 and IN and Dynamic Hip Screw 10.8) [2]. In comparison our values were higher with an average of 15 points. The improvement obtained in the patients of Guzik et al. is remarkable [2], 13 points for the HM group and 7 points in the IN group at 14-days after surgery follow-up; in comparison, in our study we recorded an improvement of only one point in the IN group and even an initial worsening in the HM group of 3 points at the first follow-up. Both group in our study reached a MSTS similar to Guzik’s patients only at the 1-year follow-up. In any case, the results were in line with what Janssen et al. reported in their systematic review on this field [48].

Analyzing VAS scale and MSTS trends is possible to notice how the IN group rapidly achieves a greater functional recovery in the first month after surgery, but this advantage is lost in the following months, so much so that from the 6 months follow-up after surgery the HM group reaches more stable and satisfactory values. In this context, we must also take into account the choice of our institution to use uncemented megaprostheses that allow a more gradually improvement trends respect uncemented ones [25].

Regardless of the functional results, surgery of the proximal femur metastases is burdened by a high rate of complications [44, 49, 50]. In the study of Meynard et al. is specified that there are no statistically significant differences in the incidence of complications between HM and IN [40], which is also confirmed by our data. In particular, compared to what reported by Wedin et al. [41], the rate of dislocations (13.8%), periprosthetic fractures (3.7%) and osteosynthesis failures (16.2%) was found to be much lower (respectively by 4%, 0% and 5%). Meynard et al. demonstrated a surgical site infection rate of 4.3% in the HM group and 1.4% in the IN group [40], but in our work we find a 5% incidence in the IN group and no cases of infection in the HM group; this difference is probably due to the small sample size. Another important factor to consider influencing incidence of complications was the use of Trevira Tube (Implantcast) and silver-coated prostheses in all HM cases. The use of the Trevira Tube has been shown to be safe and effective in reducing the dislocation rate [26, 27]. Likewise, the use of silver-coated prostheses guarantees a reduction of post-operative infections, especially early-infections [44, 51, 52]. In light of the above, the reduced complication rate especially dislocations and infections in the HM group could be partly explained by the use of the Trevira Tube and silver-coated prostheses. The possible meaning of any membranes induced by Trevira Tube or silver remains to be understood [53]. In any case, the results were in line with what Janssen et al. reported in their systematic review on this field [48]. We did not find important predictors of postoperative complications, as in other studies and fields [43, 44, 49, 54], probably due to the small sample available.

Our study has several limitations: first of all, the sample was limited for both populations examined, this is also due to stringent inclusion criteria and rarity of the pathologies; another limitation is represented by the reduced follow-up and the lack of survival analysis of the implants and patients; the comorbidities present in patients that could alter the outcomes were not examined; the study design is retrospective.

However, our study also has numerous strengths: first of all, the study design was planned to minimize any bias, the work was focused only on metastases of the trochanteric region, a single intramedullary nail and megaprosthesis design was used; only three experienced surgeons operated all patients; our work is one of the few that studies the post-operative trend of clinical function scores and therefore allows us to derive important information. Finally, to the knowledge of the authors, this is the first work to consider NLR and PLR in patients with proximal femur metastases. NLR and PLR could be used as prognostic factors to evaluate the surgical choice and it could be interesting to explore other metabolic fields, such as the Euthyroid Sick Syndrome [55, 56], in order to better stratify the population and choose the most appropriate surgical treatment.

## Conclusion

The treatment of metastases of the trochanteric region of the femur is still debated in the literature. Our study compared the functional outcomes and complications among patients undergoing intramedullary nailing or resection and megaprosthesis implantation. The data suggest that intramedullary nailing guarantees a rapid functional recovery which however gradually decreases over time, compared to patients undergoing hip megaprosthesis who instead improve gradually over time. The complications are comparable in the two groups. The selection of patients with poor prognosis allows the correct surgical indication of intramedullary nailing, while in the case of a more favorable prognosis, the intervention of hip megaprosthesis is to be preferred.

## Data Availability

The datasets used and/or analyzed during the current study are available from the corresponding author on reasonable request.

## Abbreviations

IN: Intramedullary nailing
HM: Hip megaprosthesis
NLR: Neutrophil–lymphocyte ratio
PLR: Platelet-lymphocyte ratio
MSTS: Musculoskeletal tumor society
VAS: Visual analogue scale
